# Sex-Related Differences in Protein Expression in Sarcomere Mutation-Positive Hypertrophic Cardiomyopathy

**DOI:** 10.3389/fcvm.2021.612215

**Published:** 2021-03-01

**Authors:** Maike Schuldt, Larissa M. Dorsch, Jaco C. Knol, Thang V. Pham, Tim Schelfhorst, Sander R. Piersma, Cris dos Remedios, Michelle Michels, Connie R. Jimenez, Diederik W. D. Kuster, Jolanda van der Velden

**Affiliations:** ^1^Amsterdam UMC, Department of Physiology, Amsterdam Cardiovascular Sciences, Vrije Universiteit Amsterdam, Amsterdam, Netherlands; ^2^Amsterdam UMC, Department of Medical Oncology, OncoProteomics Laboratory, VUmc-Cancer Center Amsterdam, Vrije Universiteit Amsterdam, Amsterdam, Netherlands; ^3^Victor Chang Cardiac Research Institute, Darlinghurst Sydney, Sydney, NSW, Australia; ^4^Sydney Heart Bank, Discipline of Anatomy, Bosch Institute, University of Sydney, Sydney, NSW, Australia; ^5^Department of Cardiology, Thorax Center, Erasmus Medical Center Rotterdam, Rotterdam, Netherlands

**Keywords:** hypertrophic cardiomyopathy, proteomics, sex-differences, tubulin, heat shock proteins, tissue samples

## Abstract

**Background:** Sex-differences in clinical presentation contribute to the phenotypic heterogeneity of hypertrophic cardiomyopathy (HCM) patients. While disease prevalence is higher in men, women present with more severe diastolic dysfunction and worse survival. Until today, little is known about the cellular differences underlying sex-differences in clinical presentation.

**Methods:** To define sex-differences at the protein level, we performed a proteomic analysis in cardiac tissue obtained during myectomy surgery to relieve left ventricular outflow tract obstruction of age-matched female and male HCM patients harboring a sarcomere mutation (*n* = 13 in both groups). Furthermore, these samples were compared to 8 non-failing controls. Women presented with more severe diastolic dysfunction.

**Results:** Out of 2099 quantified proteins, direct comparison of male, and female HCM samples revealed only 46 significantly differentially expressed proteins. Increased levels of tubulin and heat shock proteins were observed in female compared to male HCM patients. Western blot analyses confirmed higher levels of tubulin in female HCM samples. In addition, proteins involved in carbohydrate metabolism were significantly lower in female compared to male samples. Furthermore, we found lower levels of translational proteins specifically in male HCM samples. The disease-specificity of these changes were confirmed by a second analysis in which we compared female and male samples separately to non-failing control samples. Transcription factor analysis showed that sex hormone-dependent transcription factors may contribute to differential protein expression, but do not explain the majority of protein changes observed between male and female HCM samples.

**Conclusion:** In conclusion, based on our proteomics analyses we propose that increased levels of tubulin partly underlie more severe diastolic dysfunction in women compared to men. Since heat shock proteins have cardioprotective effects, elevated levels of heat shock proteins in females may contribute to later disease onset in woman, while reduced protein turnover in men may lead to the accumulation of damaged proteins which in turn affects proper cellular function.

## Introduction

Hypertrophic cardiomyopathy (HCM) is the most prevalent inherited cardiac disease with a prevalence of 1:200-500 (1, 2). Clinically, it is characterized by unexplained asymmetric left ventricular hypertrophy and diastolic dysfunction (3, 4). A pathogenic mutation is identified in about 50–60% of all patients (5). Both genetic and clinical heterogeneity is large, with more than 1,500 identified gene mutations, and mutation carriers who are asymptomatic, die of acute cardiac arrest or show end-stage heart failure (6).

Sex-differences in clinical presentation contribute to the phenotypic heterogeneity of HCM. Several large cohort studies observed a higher disease prevalence in men representing 55–65% of the total HCM population (7–11). At HCM diagnosis, women are on average 9 years older than men (7, 12), and at the time of myectomy surgery women are on average 7 years older than men (13). While women display less ventricular remodeling (14, 15), several studies have demonstrated more severe diastolic dysfunction (13, 16–18) and worse survival compared to men (19). Based on recent studies, our group put forward the hypothesis that disease severity in female patients with HCM is underestimated because females have smaller hearts than men, and the diagnostic criterion of ≥15 mm wall thickness does not take into account a correction by body surface area (BSA) (20). Consequently, women may be diagnosed at a later disease stage, since it takes more time for them to reach the diagnostic threshold of 15 mm wall thickness. This is supported by the observation that differences in wall thickness between genotype-positive men and women, that presented for cardiac screening, are mitigated after correction for BSA (21).

Recently, efforts have been made to understand the sex-specific phenotypical differences on a cellular level. Single cardiomyocyte studies did not observe sex-specific changes in passive stiffness compared to non-failing controls, and the HCM-related increase in myofilament Ca^2+^-sensitivity was similar in male and female HCM patients, implying that the sex-difference in diastolic dysfunction is not explained by sarcomere function itself (13). However, women had more fibrosis compared to men, expressed more compliant titin and showed reduced levels of calcium-handling proteins (13). These findings are first indications of differences between males and females on a cellular level. To further investigate cellular alterations that may underlie the sex-differences in HCM, we analyzed protein expression data of males and females in a proteomics data set of septal myocardial tissue that was collected during myectomy surgery.

By direct comparison of male and female samples we found higher levels of tubulin subunits and heat shock proteins in females. The levels of α-tubulin, determined by Western blot analysis, correlate with diastolic function displayed as E/e' and may therefore at least partly underlie the sex-difference in diastolic dysfunction. The increased levels of heat shock proteins in females are proposed to be cardioprotective (22, 23) and may contribute to later disease onset in women.

## Methods

### Proteomics Analysis

The proteomics data in this study is a new analysis of a subset of the samples that were included of the study from Schuldt et al. (24), where we identified HCM-specific protein changes compared to non-failing controls. We here focus on sex-specific protein changes in HCM by comparing cardiac samples from age-matched female and male genotype-positive HCM patients. In addition, a comparison was made between the proteomics data from the female and the male group, and the proteomic data from the non-failing control group. HCM patient tissue from the interventricular septum (IVS) of HCM patients was obtained during myectomy surgery to relieve left ventricular outflow tract obstruction (LVOTO). The study protocol for the human tissue samples was approved by the local medical ethics review committees and written informed consent was obtained from each patient prior to surgery.

For the analysis of sex-differences age-matched sarcomere mutation-positive (SMP) female (*n* = 13) and male (*n* = 13) samples were compared in a group-wise comparison using the beta binomial test as described previously (24, 25). Furthermore, the male and female SMP samples were compared to 8 non-failing healthy controls (NF_IVS_; 5 females and 3 males) obtained from the Sydney Heart Bank (HREC Univ Sydney 2012/030). The non-failing controls have no history of cardiac disease and do not take any medication. The clinical characteristics of the groups are summarized in Table 1, individual patient characteristics are displayed in Supplementary Table 1.

**Table 1:** Clinical characteristics.

	**HCM_**female**_**	**HCM_**male**_**	***P***
	**(*n* = 13)**	**(*n* = 13)**	
Age at myectomy (years)	48.5 ± 17.7	49.8 ± 15.5	0.85
LVOTg (mmHg)	60.4 ± 31.8	55.6 ± 32.2	0.72
**LV PARAMETERS**			
LAD (mm)	45.5 ± 4.3	48.4 ± 7.3	0.25
IVS (mm)	21.0 [20.0–23.0]	21.0 [18.3–23.0]	0.80
IVS_i_	12.5 [10.0–13.8]	10.0 [8.3–10.8]	0.07
EDD (mm)	41.5 [39.3–42.8]	43.5 [40.0–46.5]	0.39
ESD (mm)	16.7 ± 1.2	24.9 ± 4.7	*0.02
**SYSTOLIC PARAMETER**			
FS (%)	56.7 ± 2.5	44.4 ± 12.3	0.13
**DIASTOLIC PARAMETERS**			
E/A ratio	1.20 [0.79–2.17]	0.87 [0.74–1.40]	0.34
E/e' ratio	20.3 [17.9–32.7]	13.9 [12.6–16.0]	***0.0003
TR velocity (cm/s)	2.6 [2.1–2.9]	2.3 [2.2–2.4]	0.20
**GRADE OF DIASTOLIC DYSFUNCTION**			
1	11.1% (1)	75.0% (9)	**0.008
2	55.6% (5)	8.3% (1)	*0.046
3	33.3% (3)	16.7% (2)	0.61
**MEDICATION**			
beta blocker	84.6% (11)	84.6% (11)	>0.9999
calcium channel blocker	46.2% (6)	23.1% (3)	0.41
statins	15.4% (2)	23.1% (3)	>0.9999

*Table displays clinical characteristics of female and male HCM patient group with testing for statistical differences. LVOTg, left ventricular outflow tract gradient; LAD, left atrial diameter; IVS, interventricular septum; IVS_i_, indexed interventricular septum thickness corrected for body surface area (BSA); EDD, end-diastolic diameter; ESD, end-systolic diameter; FS, fractional shortening*.

### Tissue Homogenization

Pulverized frozen tissue was homogenized in 40 μl/mg tissue 1x reducing sample buffer (106 mM Tris-HCl, 141 mM Tris-base, 2% lithium dodecyl sulfate (LDS), 10% glycerol, 0.51 mM EDTA, 0.22 mM SERVA Blue G250, 0.18 mM Phenol Red, 100 mM DTT) using a glass tissue grinder. Proteins were denatured by heating to 99°C for 5 min, after which samples were sonicated and heated again. Debris was removed by centrifugation at maximum speed for 10 min in a microcentrifuge (Sigma, 1-15K).

### Protein Fractionation

Proteins were separated using 1D SDS-PAGE. Samples from each group were loaded alternating on the gels to avoid bias. Equal volumes of sample (30 μl protein homogenate per sample, containing ~20–30 μg of protein) were loaded on a precast 4–12% NuPAGE Novex Bis-Tris 1.5 mm mini gel (Invitrogen). Electrophoresis was performed at 200V in NuPAGE MES SDS running buffer until the dye front reached the bottom of the gel. Gels were fixed in a solution of 50% ethanol and 3% phosphoric acid, and stained with 0.1% (w/v) Coomassie brilliant blue G-250 solution (containing 34% (v/v) methanol, 3% (v/v) phosphoric acid and 15% (w/v) ammonium sulfate).

### In-Gel-Digestion

In-gel digestion was performed as described previously (26). The proteins were in-gel reduced with 10 mM DTT and alkylated with 54 mM iodoacetamide. Each gel lane was cut into 5 pieces which were subsequently sliced into 1 mm3 cubes. Proteins were digested in-gel with 6.3 ng/ml trypsin. Peptides were extracted from gel slices with 1% formic acid and 5% formic acid/50% acetonitrile and concentrated in a vacuum centrifuge prior to nano-LC-MS/MS measurement. Samples were measured by LC-MS per gel band starting at the high molecular weight (MW) fraction for all samples, before continuing with the next gel band until the last (low MW fraction) band was measured. Injections alternated between all different group samples to minimize experimental bias between groups.

### Nano-LC-MS/MS

Analysis of the experiment was performed as described in Piersma et al. (27). Peptides were separated using an Ultimate 3000 Nano LC-MS/MS system (Dionex LC-Packings, Amsterdam, The Netherlands) equipped with a 40 cm × 75 μm ID fused silica column custom packed with 1.9 μm,120 Å ReproSil Pur C18 aqua (Dr. Maisch GMBH, Ammerbuch-Entringen, Germany). After injection, peptides were trapped at 6 μl/min on a 10 mm × 100 μm ID trap column packed with 5 μm, 120 Å ReproSil Pur C18aqua at 2% buffer B (buffer A: 0.5% acetic acid (Fischer Scientific), buffer B: 80% acetonitrile, 0.5% acetic acid) and separated at 300 nl/min in a 10–40% buffer B gradient in 60 min (90 min inject-to-inject). Eluting peptides were ionized at a potential of + 2 kV into a Q Exactive mass spectrometer (Thermo Fisher, Bremen, Germany). Intact masses were measured at resolution 70,000 (at m/z 200) in the orbitrap using an automatic gain control (AGC) target value of 3 × 10^6^ charges. The top 10 peptide signals (charge-states 2+ and higher) were submitted to MS/MS in the HCD (higher-energy collision) cell using 1.6 amu isolation width and 25% normalized collision energy. MS/MS spectra were acquired at resolution 17,500 (at m/z 200) in the orbitrap using an AGC target value of 1 × 10^6^ charges, a maxIT of 60 ms and an underfill ratio of 0.1%. Dynamic exclusion was applied with a repeat count of 1 and an exclusion time of 30 s.

### Data Analysis

MS/MS spectra were searched against a Uniprot human reference proteome FASTA file (Swissprot_2017_03_human_canonical_and_isoform.fasta, 42161 entries) using MaxQuant version 1.5.4.1. Enzyme specificity was set to trypsin and up to two missed cleavages were allowed. Cysteine carboxamidomethylation was treated as fixed modification, and methionine oxidation and N-terminal acetylation as variable modifications. Peptide precursor ions were searched with a maximum mass deviation of 4.5 parts per million (ppm) and fragment ions with a maximum mass deviation of 20 ppm. Peptide and protein identifications were filtered at a false discovery rate (FDR) of 1% using the decoy database strategy. The minimal peptide length was 7 amino acids, the minimum Andromeda score for modified peptides was 40, and the minimum delta score was 6. Proteins that could not be differentiated based on MS/MS spectra alone were grouped to protein groups (default MaxQuant settings). Searches were performed with the label-free quantification option selected. The mass spectrometry proteomics data have been deposited to the ProteomeXchange Consortium via the PRIDE (28) partner repository with the dataset identifier PXD012467. Beta-binominal statistics were used to assess differential protein expression between groups, after normalization on the sum of the counts for each sample (25). Proteins with a *p* value below 0.05 were considered significantly differentially expressed. Proteins which were present in <25% of the samples or had an average normalized count of <1.4 were excluded from further functional analysis. Principal component analysis was performed in R. Therefore, quantile normalization and log2 transformation was performed on the normalized counts. The 95th Percentile was taken, the data median centered and the principal components calculated. Hierarchical clustering was performed after a statistical multi-group comparison. Protein networks were generated utilizing the STRING database (Search Tool for the Retrieval of Interacting Genes/Proteins) and visualized with Cytoscape software (29). Protein interaction networks were generated with ClusterONE and gene ontology (GO) analysis was performed using the BiNGO application in cytoscape (30, 31). Venn diagrams were created with InteractiVenn tool (32) and the layout modified if needed.

### Transcription Factor Analysis

The ToppFun tool from the ToppGene Suite was used to identify transcription factors of significantly different proteins between HCM_female_ and HCM_male_ (33). All significantly different proteins between HCM_female_ and HCM_male_ were used as input.

### Western Blot

For analysis of protein levels by Western blot, whole tissue lysates were used from either the proteomic analysis or were prepared as described previously (34). Proteins were separated on precast SDS-PAGE 4–12% criterion gels (Bio-Rad) and transferred to polyvinylidene difluoride or nitrocellulose membranes. Site-specific antibodies directed to acetylated α-tubulin (Sigma, T7451), HSPA1 (Enzo Life Sciences, ADI-SPA-810), HSPA2 (Proteintech group, 66291-1), HSPB1 (Enzo Life Sciences, ADI-SPA-800), HSPB5 (Enzo Life Sciences, ADI-SPA-223), HSPB7 (abcam, ab150390), HSPD1 (Enzo Life Sciences, ADI-SPA-805), HSPA4 (Cell Signaling, 3303S), HSP90 (Cell Signaling, 4874S), α-tubulin (Sigma, T9026), tyrosinated tubulin (Sigma, T9028), detyrosinated tubulin (abcam, ab48389), and GAPDH (Cell Signaling, 2118S; Fitzgerald, 10R-G109a) were used to detect the proteins which were visualized with an enhanced chemiluminescence detection kit (Amersham) and scanned with Amersham Imager 600. Protein levels were determined by densitometric analysis and normalized to GAPDH.

### Statistics

Graphpad Prism v8 software was used for statistical analysis. Normally distributed data (except proteomics data) were statistically analyzed with the Student's *t*-test when comparing 2 groups and with one-way ANOVA when comparing more than 2 groups. Non-normally distributed data were analyzed with Mann-Whitney test. Linear regression was statistically tested with Pearson correlation. Data are presented as means ± standard errors of the mean, clinical characteristics are presented as mean ± standard deviation or median with interquartile range when appropriate. Categorical data was statistically analyzed using Fisher's exact test and presented as frequencies. A *p* ≤ 0.05 was considered as significantly different.

## Results

**Figure 1:**
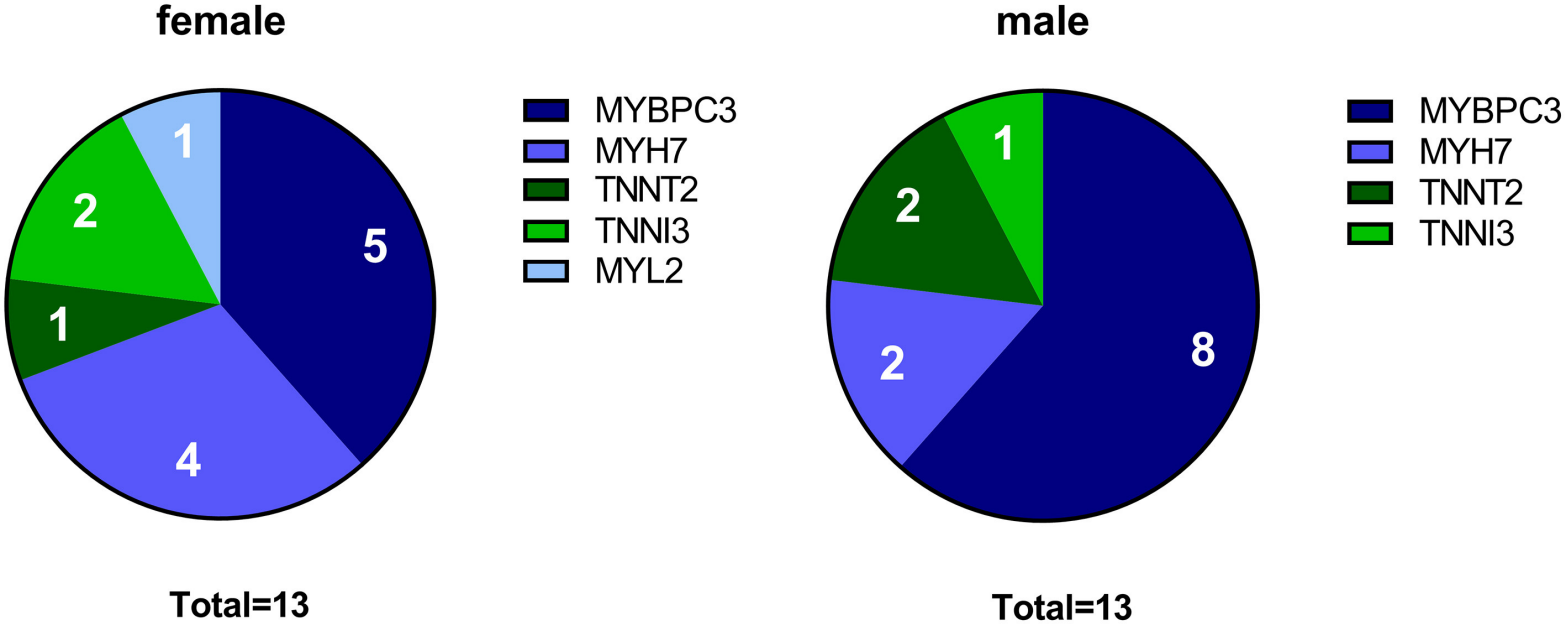
Genotypes in female and male HCM patient groups. The pie charts indicate the number of patients with a mutation in *MYBPC3, MYH7, TNNT2, TNNI3*, and *MYL2* for the female and male group, respectively.

To define sex-specific protein changes in HCM, we compared the protein expression profile of male and female HCM samples. We performed a new analysis on a subset of patients from our proteomics data set (24). We compared age-matched samples from 13 male and 13 female sarcomere mutation-positive (SMP) HCM patients. The genotype distribution of both groups is depicted in Figure 1 and shows that the majority of mutations in both groups are located in the thick filament genes *MYBPC3* and *MYH7*. Clinical characteristics of the groups are summarized in Table 1. Both male and female patients had obstructive HCM. While male patients presented with a larger LV end-systolic diameter (ESD), females displayed more severe diastolic dysfunction, indicated by a higher E/e' ratio compared to male patients, and more females with diastolic dysfunction stage 2 (35, 36). Although there is no difference between women and men in absolute IVS thickness, the women included in this study showed a higher IVS thickness when corrected for BSA compared to males (25% increase, *p* = 0.07; Table 1). This is in line with a previous study showing significant differences in IVS thickness between women and men when corrected for BSA (13).

### Females Express More Tubulin and Heat Shock Proteins

**Figure 2:**
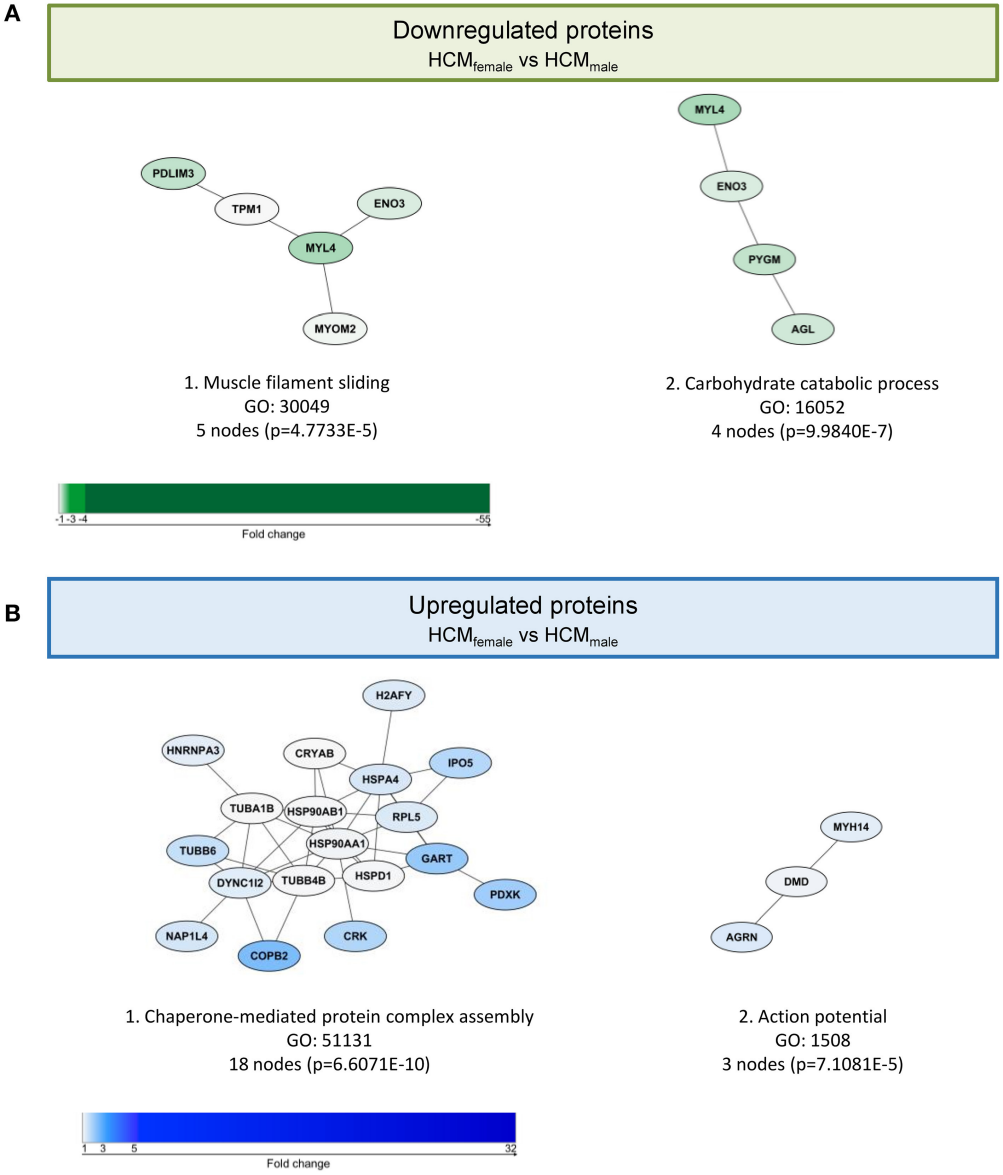
Functional protein cluster of the direct comparison of HCM_female_ and HCM_male_. **(A)** Illustrates the functional protein cluster of the proteins that are significantly lower expressed in females compared to males. **(B)** Shows the functional protein cluster of the proteins that are significantly higher expressed in females compared to males. For each protein cluster the most significant biological process is given. Proteins with a *p* <0.05 were used for the analysis.

**Figure 3:**
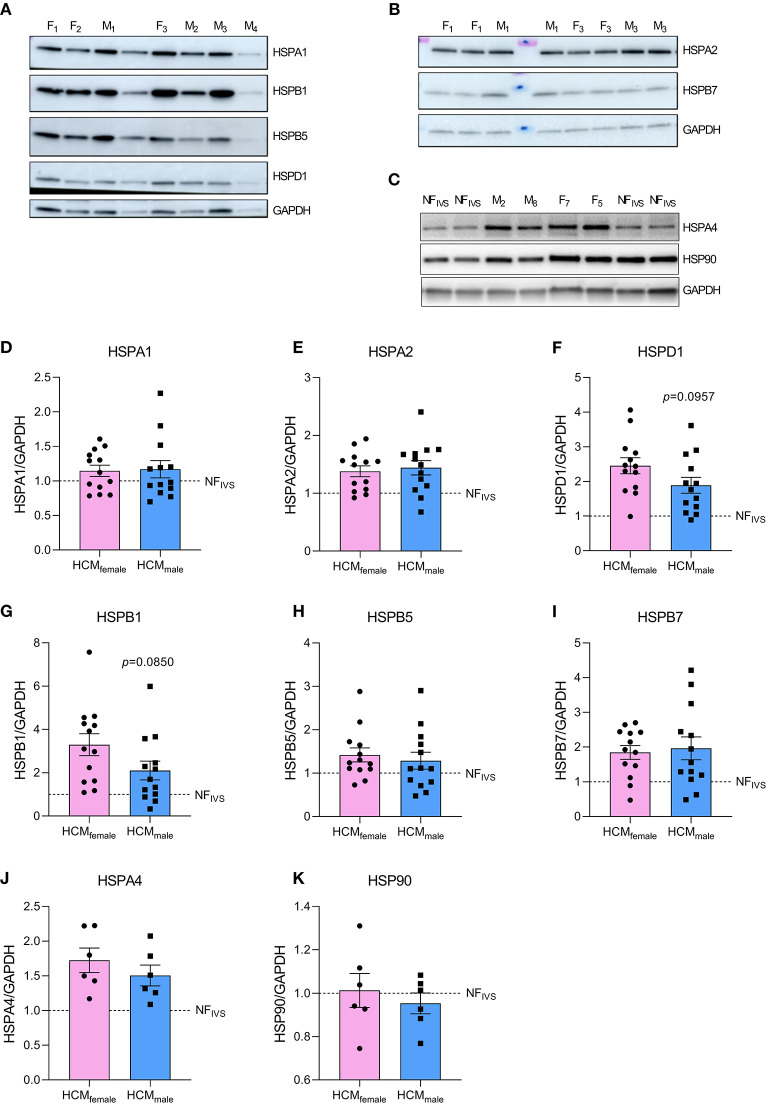
Protein levels of heat shock proteins determined by western blot. **(A–C)** Show representative western blot images. **(D–K)** Display quantified protein levels for HSPA1, HSPA2, HSPD1, HSPB1, HSPB5, HSPB7, HSPA4, and HSP90, respectively. Data were statistically analyzed by unpaired two-tailed *t*-test. Dashed line indicates protein levels in the NF_IVS_. Western blot dataset is partly derived from Dorsch et al. and re-analyzed for the age-matched samples included in the current study (34).

**Figure 4:**
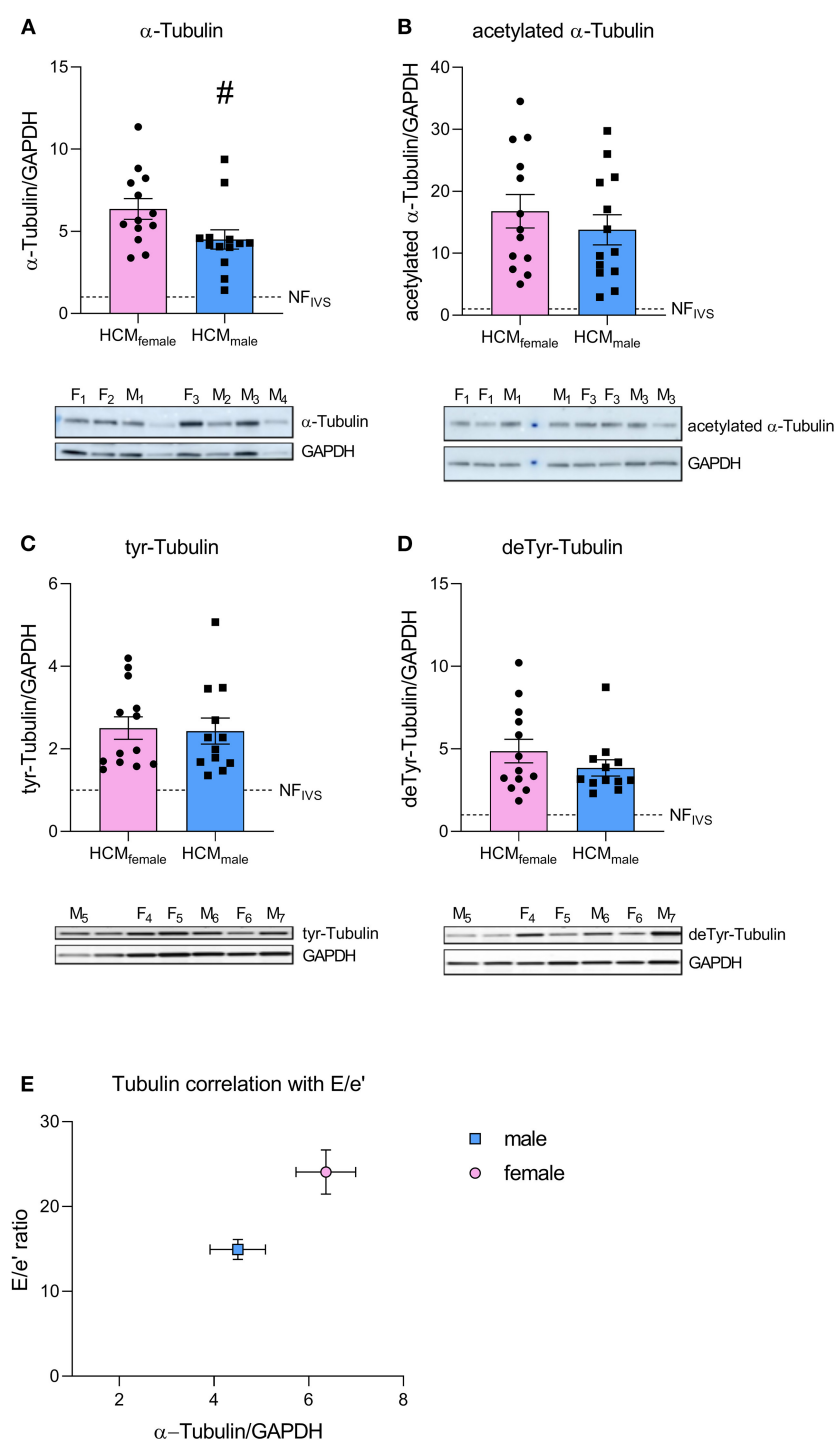
Protein levels of α-tubulin and its posttranslational modifications determined by western blot. **(A–D)** Show protein levels of α-tubulin **(A)**, acetylated tubulin **(B)**, tyrosinated tubulin **(C)** and detyrosinated tubulin **(D)** with representative images. Data were statistically analyzed by unpaired two-tailed *t*-test, ^#^*p* = 0.0407. Dashed line indicates protein levels in the NF_IVS_. Western blot dataset is new analysis of a subset of samples from Dorsch et al. and Schuldt et al. (24, 34). **(E)** Shows correlation of α-tubulin levels with E/e' ratio.

Out of 2099 quantified proteins, only 46 proteins were significantly differentially expressed in the direct comparison of the female and male group. Two functional protein interaction clusters were identified for both the 14 downregulated and the 32 upregulated proteins in females compared to males. The functional protein clusters that were less expressed in females compared to males were related to muscle filament sliding and carbohydrate catabolic process (Figure 2A). The functional protein clusters of proteins that are more expressed in females compared to males are chaperone-mediated protein complex assembly and action potential (Figure 2B). Chaperone-mediated complex assembly is, based on the number of proteins in this cluster (18, 39% of the differentially expressed proteins), the dominating protein cluster in the comparison of female and male SMP HCM samples. Interestingly, this cluster contains mainly heat shock proteins (HSPs) and tubulin subunits. HSPs and tubulin have already been investigated before by our group in the context of SMP and sarcomere-mutation negative HCM samples (24, 34). Therefore, we now further determined if there are any sex-differences at the protein levels of tubulin and a selection of HSPs that were assessed by western blot. While we did not observe significant sex-differences in the levels of HSPA1, HSPA2, HSPB5, HSPB7, HSPA4 and HSP90, we found a trend to higher levels of HSPD1 (*p* = 0.0957) and HSPB1 (*p* = 0.0850) in female compared to male samples [Figure 3, re-analyzed from the dataset from Dorsch et al. (34)]. Although the HSPs assessed by western blot do not show significant differences between females and males, the data are in line with the small fold changes found in the proteomics data and point toward higher levels of HSPs in female compared to male HCM tissue. Likewise, we analyzed the sex-differences in our tubulin data set (Figure 4), re-analyzed from the datasets from Dorsch et al. and Schuldt et al. (24, 34). In line with the proteomics data, levels of α-tubulin were significantly higher in females compared to males, whereas we did not find any sex-differences in the posttranslational modifications acetylation, tyrosination and detyrosination. To determine if elevated levels of tubulin may correlate with diastolic dysfunction, we plotted levels of α-tubulin with the clinical parameter E/e' for male and female patients separately (Figure 4E). Female patients have higher levels of α-tubulin in combination with more severe diastolic dysfunction, as indicated by increased E/e', compared to male patients who show lower levels of α-tubulin with less severe diastolic dysfunction.

### Proteins Involved in Translation Are Specifically Downregulated in Male HCM Patients Compared to Non-failing Control Myocardium

**Figure 5:**
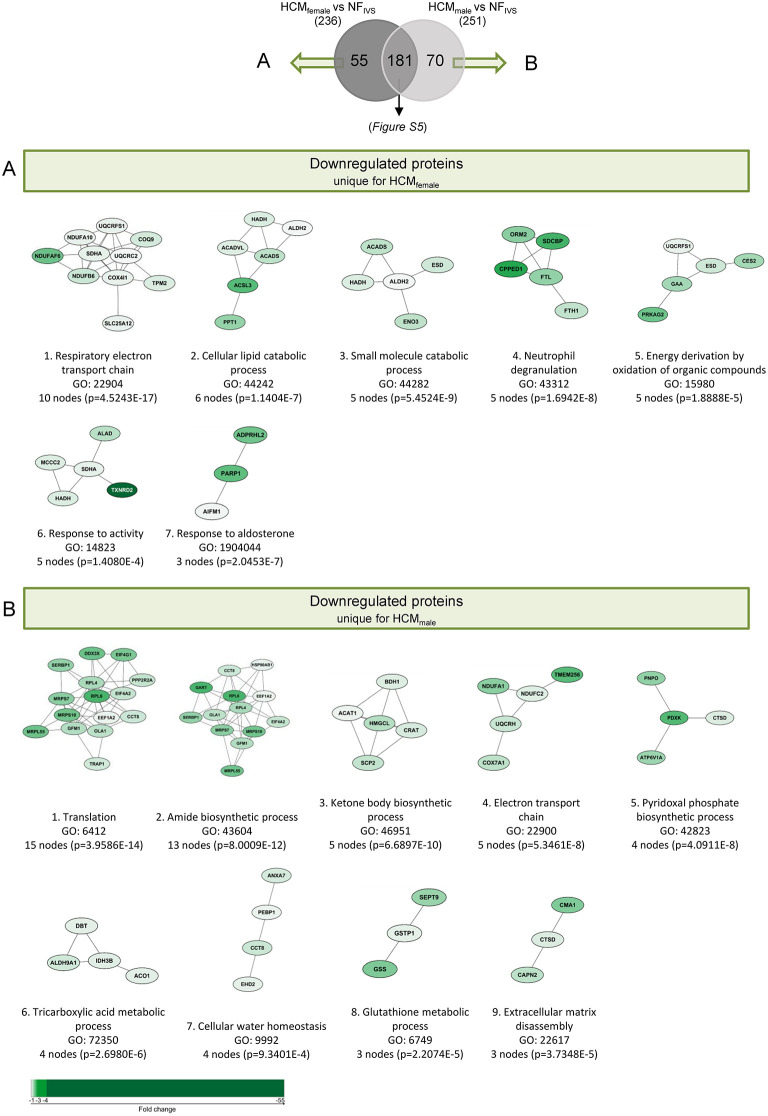
Functional protein cluster of the downregulated proteins that are unique for either HCM_female_ or HCM_male_ when compared separately to NF_IVS_. A Venn diagram was created with the significantly lower expressed proteins between HCM_female_ and NF_IVS_ and HCM_male_ and NF_IVS_, to identify the downregulated proteins that are unique for either males or females, or shared by both groups. **(A)** Illustrates the functional protein cluster of the proteins that are only significantly lower expressed in females when compared to NF_IVS_, whereas **(B)** shows the functional protein cluster of the proteins that are only significantly lower expressed in males when compared to NF_IVS_. For each protein cluster the most significant biological process is given. Proteins with a *p* < 0.05 were used for the analysis.

**Figure 6:**
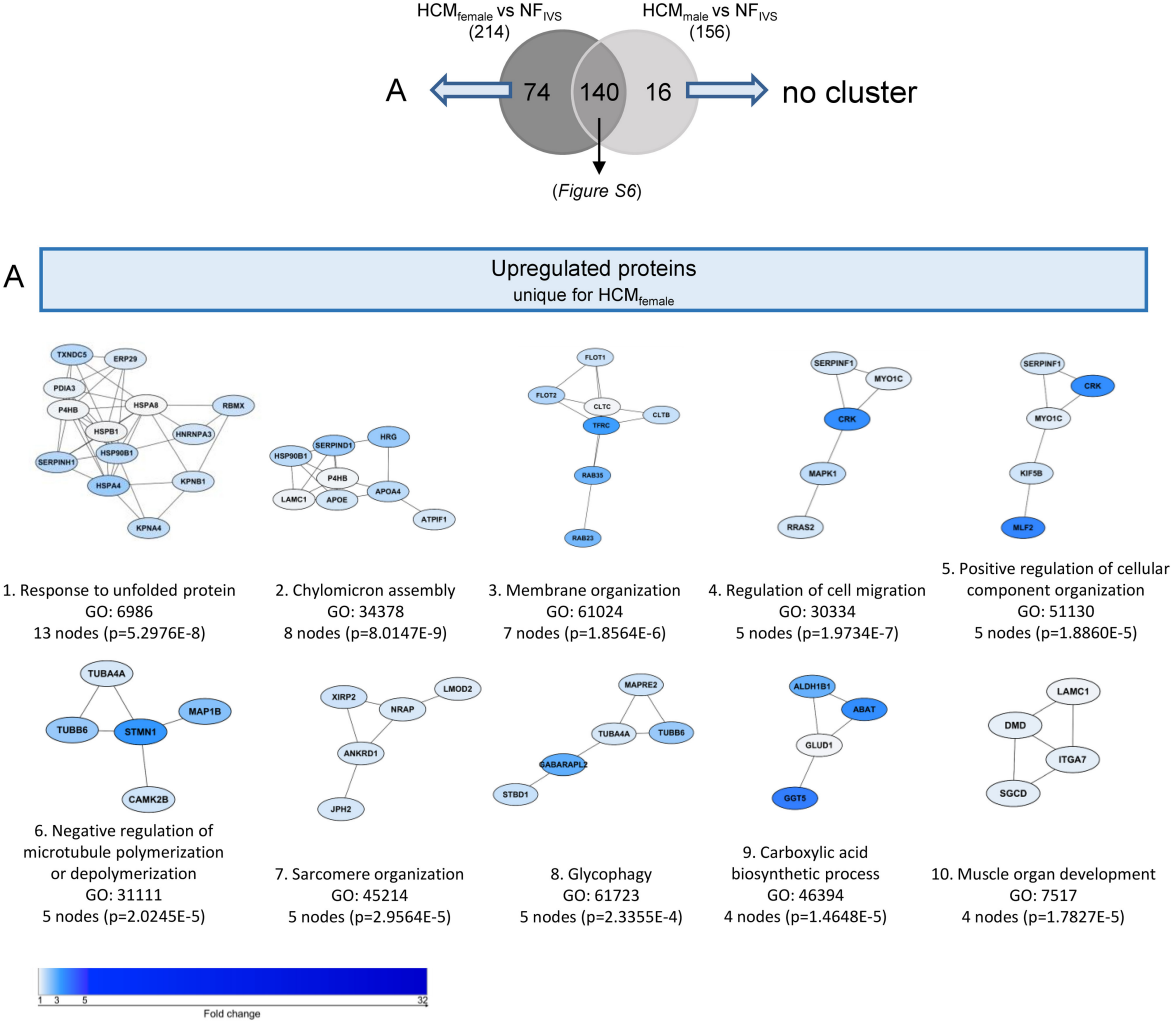
Functional protein cluster of the upregulated proteins that are unique for either HCM_female_ or HCM_male_ when compared separately to NF_IVS_. A venn diagram was created with the significantly higher expressed proteins between HCM_female_ and NF_IVS_ and HCM_male_ and NF_IVS_, to identify the upregulated proteins that are unique for either males or females or shared by both groups. **(A)** Illustrates the functional protein cluster of the proteins that are only significantly higher expressed in females when compared to NF_IVS_. The proteins only significant for HCM_male_ did not give any functional protein cluster. For each protein cluster the most significant biological process is given. Proteins with a *p* < 0.05 were used for the analysis.

As an additional approach, we compared the protein expression of both the male and the female group to NF_IVS_ to look at specific sex-related protein changes compared to non-failing myocardium, thereby identifying proteins that are not only sex- but also disease-specific. We have identified 236 proteins that are less expressed and 214 proteins that are more expressed in the female HCM patients vs. NF_IVS_. In the male HCM patients we identified 251 lower expressed proteins and 156 more abundant proteins compared to NF_IVS_. The top 10 functional protein interaction clusters of the females compared to NF_IVS_ are listed in Table 2 and the top 10 protein clusters of the male samples compared to NF_IVS_ are displayed in Table 3. The complete set of protein interaction clusters resulting from this analysis is shown in Supplementary Figures 1–4. To identify differences between female and male HCM using this analysis approach, we created Venn diagrams of the significantly different proteins from the female vs. NF_IVS_ and the male vs. NF_IVS_ comparison to look at overlapping proteins that are shared by both comparisons, and proteins that are unique for either males or females compared to NF_IVS_ (Figures 5, 6, Venn diagrams).

**Table 2:** Top 10 differentially regulated pathways in HCM_female_ vs. NF_IVS_.

**Downregulated proteins**			**Upregulated proteins**		
**Pathway (GO ID)**	**Nodes**	***p***	**Pathway (GO ID)**	**Nodes**	***p***
Cellular respiration (45333)	70	6.7945E-73	Extracellular structure organization (43062)	45	8.1156E-43
NAD metabolic process (19674)	28	1.7205E-22	Muscle contraction (6936)	19	1.9361E-16
Monocarboxylic acid metabolic process (32787)	22	2.3313E-25	Post-translational protein modification (43687)	17	1.4103E-11
Organic acid catabolic process (16054)	20	1.0701E-24	Organelle localization (51640)	13	1.6284E-8
Neutrophil degranulation (43312)	15	2.5257E-15	Response to unfolded protein (6986)	12	2.2706E-12
Cellular nitrogen compound biosynthetic process (44271)	12	3.4079E-11	Striated muscle cell development (55002)	12	6.9444E-9
Muscle contraction (6936)	12	5.6834E-9	Myofibril assembly (30239)	10	4.2003E-14
Acute-phase response (6953)	11	5.0201E-14	Regulation of cell migration (30334)	9	1.3513-8
Cellular detoxification (1990748)	9	6.5002E-10	Cellular carbohydrate metabolic process (44262)	8	2.0038E-9
Cardiac muscle tissue development (48738)	8	9.5592E-12	Membrane organization (61024)	8	4.7889E-6

*Table lists the top 10 functional protein cluster (based on size) of the down- and upregulated proteins for the comparison of HCM_female_ with NF_IVS_. Displayed are the most significant biological process (gene ontology), the number of nodes in the cluster and the p-value*.

**Table 3:** Top 10 differentially regulated pathways in HCM_male_ vs. NF_IVS_.

**Downregulated proteins**			**Upregulated proteins**		
**Pathway (GO ID)**	**Nodes**	***p***	**Pathway (GO ID)**	**Nodes**	***p***
Cellular respiration (45333)	62	9.9733E-62	Extracellular matrix organization (30198)	47	9.0957E-40
Small molecule metabolic process (44281)	29	3.0796E-22	Actin filament-based process (30029)	13	3.2873E-10
Amide biosynthetic process (43604)	27	2.6386E-19	Striated muscle cell development (55002)	12	4.8955E-11
Carboxylic acid catabolic process (46395)	26	3.3911E-26	Platelet aggregation (70527)	11	1.0179E-8
Regulated exocytosis (45055)	15	3.5280E-13	Muscle contraction (6936)	10	1.1585E-7
Translational initiation (6413)	15	6.4097E-8	Calcium-independent cell-matrix adhesion (7161)	8	5.7263E-7
Protein folding (6457)	15	1.9000E-7	Response to muscle inactivity involved in regulation of muscle adaptation (14877)	8	8.5753E-6
Organic acid catabolic process (16054)	13	1.0083E-14	Neutrophil degranulation (43312)	7	3.3963E-7
Acute-phase response (6953)	12	1.0023E-13	Carbohydrate metabolic process (5975)	6	6.4278E-8
Creatine metabolic process (6600)	12	3.2195E-12	Regulation of podosome assembly (71801)	6	9.2900E-6

*Table lists the top 10 functional protein cluster (based on size) of the down- and upregulated proteins for the comparison of HCM_male_ with NF_IVS_. Displayed are the most significant biological process (gene ontology), the number of nodes in the cluster and the p-value*.

Of the proteins that are downregulated compared to NF_IVS_ we identified 181 proteins that are overlapping between males and females. These proteins belong mainly to metabolic pathways (Supplementary Figure 5), and can be considered as general HCM-specific protein changes. Fifty five of the downregulated proteins are only significantly different for the females. The functional protein clusters of these proteins are related to respiratory electron transport chain, cellular lipid catabolic process, response to activity, small molecule catabolic process, neutrophil degranulation, energy deprivation by oxidation of organic compounds and response to aldosterone (Figure 5A). Seventy downregulated proteins are only significantly different in the male group and cluster analysis revealed that the biggest protein cluster is related to the biological process translation. It contains many ribosomal proteins that have a consistently high fold-change compared no NF_IVS_. Other clusters are related to amide biosynthetic process and several processes related to energy metabolism like ketone body biosynthetic process, electron transport chain and tricarboxylic acid metabolic process. Furthermore, we obtained protein clusters related to pyridoxal phosphate biosynthetic process, cellular water homeostasis, glutathione metabolic process, and extracellular matrix disassembly (Figure 5B).

### Microtubular and Heat Shock Proteins Are Specifically Upregulated in Female HCM Patients When Compared to Controls

**Figure 7:**
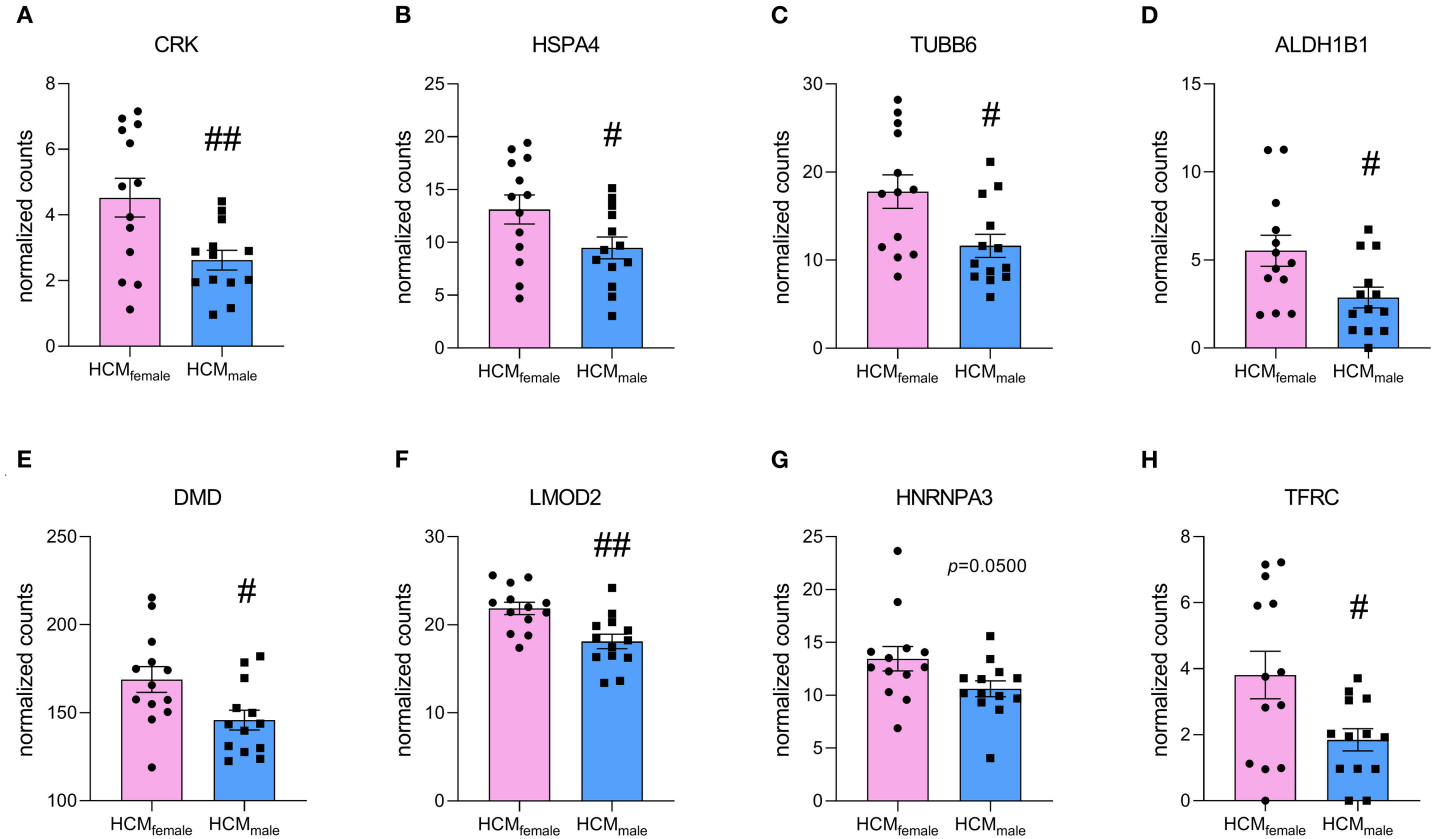
Normalized counts of proteins that are significantly upregulated in HCM_female_ compared to HCM_male_ and are uniquely upregulated in females when compared to NF_IVS_. **(A)** CRK, **(B)** HSPA4, **(C)** TUBB6, **(D)** ALDH1B1, **(E)** DMD, **(F)** LMOD2, **(G)** HNRNPA3 and **(H)** TFRC. ^#^*p* < 0.05, ^##^*p* < 0.01, unpaired two-tailed *t*-test.

For the upregulated proteins, 140 proteins are significantly more expressed in both females vs. NF_IVS_ and males vs. NF_IVS_. The functional protein interaction clusters are illustrated in Supplementary Figure 6. The 74 proteins that are significantly higher in females compared to controls result in clusters related to response to unfolded protein, chylomicron assembly, membrane organization, regulation of cell migration, negative regulation of microtubule polymerization or depolymerization, positive regulation of cellular component organization, sarcomere organization, glycophagy, muscle organ development, and carboxylic acid biosynthetic process (Figure 6A). With the cluster response to unfolded protein and negative regulation of microtubule polymerization or depolymerization the results of this approach are in line with the direct comparison of female and male HCM samples in which these proteins were represented by the cluster chaperone-mediated protein complex assembly. Eight of the 74 proteins that are significantly higher expressed only in females (CRK, HSPA4, TUBB6, ALDH1B1, DMD, LMOD2, HNRNPA3, and TFRC) overlap with the 46 proteins significantly different in the direct male and female comparison and may represent important candidates to define the female group (Figure 7).

The 16 proteins that are only significantly higher in HCM males compared to NF_IVS_ did not form any functional protein cluster. Of these proteins, only the protein SLC27A6 (solute carrier family 27 member 6/long chain fatty acid transport protein 6) overlaps with the 46 proteins that are significantly different in the direct comparison of females and males. As being significantly higher expressed compared to both NF_IVS_ and females, SLC27A6, involved in long chain fatty acid uptake, may be an important candidate defining the male HCM patients.

### Transcriptional Regulation of Significantly Different Proteins Between HCM_female_ and HCM_male_ Is Not Dominated by Sex Hormones

Since sex hormones also act as transcription factors, we analyzed if sex-hormone-related transcription factors can be responsible for the 46 significantly differentially expressed proteins of the direct comparison between female and male HCM samples (Figure 2). Possible transcription factor bindings sites were analyzed with the ToppFun database. Three hundred seventy two transcription factors were identified that have binding-sites in the 46 proteins that are significantly different between female and male HCM patient samples. The 10 most significant transcription factors are shown in Supplementary Table 2. Of these we found 7 to be sex-hormone related (Supplementary Table 3). These 7 transcription factors are involved in the regulation of 5 significantly different proteins between male and female HCM myocardium: HSPD1, PYGM, DMD, ENO3, and TFRC. Of these, DMD, ENO3, and TFRC are also significantly different when comparing females with NF_IVS_ (Figures 5, 6). With PYGM and ENO3 involved in carbohydrate metabolism, DMD involved in muscle organization and HSPD1 as a member of the PQC, we thereby identified candidate proteins that may be regulated by sex hormone-related transcription factors. However, the bioinformatical analysis revealed that the 5 proteins also have binding sites for another 258 of the 372 identified transcription factors. Based on this we can conclude that the differences found between female and male samples may be partly due to transcriptional regulation by sex hormones but that this is not the dominant mechanism driving sex-dependent differential protein expression.

## Discussion

In this study we analyzed sex-differences at the protein level in proteomics data of HCM patient tissue to define sex-specific protein expression which may explain the difference in clinical presentation. The direct comparison of the male and female patient groups resulted in only a small number of significantly different proteins. Compared to males, female HCM patients display lower levels of myofilament proteins related to the biological process muscle filament sliding and have elevated levels of tubulin and HSPs. Consistently, by subtracting the baseline differences using an additional non-failing control group, we observed a more profound elevation of HSPs and the microtubule processes in female HCM patients.

### Set of 8 Proteins Consistently Defines the Female HCM Patients

A study investigating sex-differences in gene expression at the mRNA level in idiopathic cardiomyopathy patients found 1837 differently expressed genes between male and female patients, of which the large majority of 1377 genes had a fold change <1.2 (37). Considering these low fold changes on a gene expression level, little changes at the protein level are to be expected. We found expression levels of 8 proteins significantly higher in females when directly compared to males, and also uniquely significant for females when compared to NF_IVS_ (Figure 7). Thereby, these proteins are disease-specific and may play an important role in defining the female patient group. One of these proteins is CRK, which is an adaptor protein with SH2 and SH3 domains but no catalytic region. It functions in signal transduction processes and has been shown to be involved in cardiac development (38). HSPA4 is a heat shock protein that acts as nucleotide exchange factor for HSP70 chaperones. Its expression is upregulated in response to pressure overload and in human heart failure, which is thought to be a beneficial response as it helps degrading misfolded proteins (39). TUBB6 is a β-tubulin subtype that forms microtubules together with α-tubulin. Elevated levels of tubulin have been observed in heart failure (40). ALDH1B1 is an aldehyde dehydrogenase that is involved in alcohol metabolism and has been shown to function in glucose metabolism (41). DMD encodes for dystrophin and connects the actin cytoskeleton with the extracellular matrix. As actin cross-linker, it plays an important role for the mechanical properties of the cardiomyocyte. Elevated levels in females may be explained by its gene location on the X-chromosome. Mutations in DMD have been associated with muscular dystrophies and X-linked dilated cardiomyopathy (42). LMOD2 is the cardiac isoform of leiomodin, an actin-binding protein involved in thin filament assembly. Studies in adult mice have shown that LMOD2 has an essential role in maintaining proper cardiac thin filament length and coinciding force generation (43). HNRNPA3 is a heterogeneous nuclear ribonucleoprotein that binds to single-stranded telomeric repeats to stabilize them (44), and TFRC encodes for the transferrin receptor that promotes iron uptake (45).

### Elevated Levels of HSPs and Tubulin Are in Line With a More Severe Phenotype

The most prominent difference in our study were higher levels of tubulin and HSPs in female compared to male HCM patient samples taken at the time of myectomy. Interestingly, sex-differences in the expression of HSPs have been observed before. Higher levels of HSPA1A have been measured in healthy female rat hearts compared to male, and these increased levels were estrogen dependent (46). We also found by bio-informatical analysis that HSPD1, which is elevated in female compared to male HCM myocardium, can be regulated by the estrogen-related receptor α. It may be speculated that the estrogen-dependent HSP induction, that is known to be cardioprotective (47), has also beneficial effects in HCM, leading to lower disease penetrance and later disease onset in women. Indeed, HSP activation has been shown to have beneficial effects on heart function in a mouse model of desmin-related cardiomyopathy in which HSP expression was induced by geranylgeranylacetone (48). In our study we observed increased protein expression of HSPs in tissue samples from symptomatic stage II HCM patients, which raises the question if the optimal therapeutic window for HSP induction might already be at an earlier preclinical stage of the disease. This warrants further studies in iPSC-derived human heart models and other cardiomyopathy animal models.

Cytoskeletal proteins like tubulin and desmin are known to be elevated in heart failure (40). Our group has previously shown that tubulin protein levels are increased in HCM myectomy tissue compared to NF_IVS_ (24, 34). Interestingly, tubulin and desmin protein content has been shown to correlate well with left ventricular end-diastolic pressure (LVEDP) in heart failure (40). As increased LVEDP indicates diastolic dysfunction, cytoskeletal protein levels correlate directly with diastolic dysfunction. Furthermore, tubulin has in combination with its posttranslational modifications a direct effect on contractile function, as detyrosinated tubulin binds to desmin and causes a stiffening of the myofilament (49–51). As increased levels of tubulin in females compared to males correlate with more severe diastolic dysfunction (13), proof-of-concept studies in model systems have to show whether this is a causal relationship during HCM development.

### Male Samples Display Lower Levels of Translational Proteins

Ribosomal and protein synthesis-related proteins, which form the cluster “translation,” were significantly downregulated in male HCM patients compared to NF_IVS_. Interestingly, it was recently shown that cMyBP-C protein synthesis and degradation rates were slowed down in induced pluripotent stem cell-derived cardiomyocytes harboring a heterozygous *MYBPC3* mutation (52). The authors proposed that cells harboring a *MYBPC3* truncating mutation may have the capacity to attain normal levels of cMyBP-C protein and thereby preserve cardiomyocyte function. On the other hand, protein turnover is needed to replace aged/damaged proteins. Thus, while reduced protein turnover may preserve protein stoichiometry and thereby cardiomyocyte function, it may lead to accumulation of damaged proteins in the sarcomere. Our data are in line with the study of Helms and colleagues, and imply reduced protein turnover in particular in male HCM hearts at the time of myectomy. Increasing protein translation rate to maintain proper protein turnover represents an attractive avenue to further explore.

### Sex-Specific Difference in Carbohydrate Metabolism

Downregulation of metabolic pathways is a general HCM disease hall mark (24, 53) and accordingly we did not find major differences in these pathways between males and females. However, females showed lower levels of proteins related to carbohydrate catabolic process (Figure 2A), including muscle associated glycogen phosphorylase (PYGM) and β-enolase (ENO3). Mutations in both of these genes have been associated with glycogen metabolism disorders (54). Consequently, reduced levels of PYGM and ENO3 impair glycogen metabolism in women. Our transcription factor analysis showed that both PYGM and ENO3 can be regulated by sex hormone transcription factors. The findings indicate that women may be less metabolically flexible to adapt to altered metabolic demand, at least during disease development. Imaging studies have shown that reduced cardiac efficiency occurs already at the early disease stage in asymptomatic male and female mutation carriers (55, 56) and may indicate that metabolic changes may be present in the very early stages before the onset of cardiac remodeling.

### More Fibrosis in Female Patients Is Not Reflected at Protein Level

It has been observed that female HCM patient samples show more fibrosis compared to male samples (13). Interestingly, this difference is not reflected at protein level. Neither in the direct comparison of female and male HCM samples, nor in the analysis compared to NF_IVS_, we observed a specific increase in extracellular matrix proteins for females. In the current proteomics analysis, increased expression of extracellular matrix proteins was a general disease hallmark, and common for both female and male group. The methodological difference between quantifying proteins involved in extracellular matrix organization in this proteomics analysis and measuring the actual fibrotic area in tissue sections as performed by Nijenkamp et al. may underlie the divergent findings.

### Study Limitations

The findings in this study are observational and provide a starting point for further validation in an independent cohort and proof-of-concept studies in disease models. Due to the limited availability of non-failing heart tissues, the male control samples used in this study are not age-matched with the male HCM samples, which may contribute to observed differences in protein expression. Furthermore, the group size of only female or male non-failing samples is too small to perform comparisons of HCM and controls of the same sex. Therefore, follow-up studies would benefit from age-matched and increased numbers of non-failing controls to differentiate between sex- and disease-specific differences in protein expression. As our findings point toward a possible role of sex hormone regulation, information about the hormonal state of the female subjects should be collected for future studies.

## Conclusion

This proteomic analysis of female and male SMP HCM tissue highlights that elevated protein levels of tubulin correlate with more severe diastolic dysfunction in females. Another aspect which warrants further research is reduced protein turnover, in particular in male HCM, which may represent an adaptive mechanism to maintain protein stoichiometry, though may also have a negative impact and result in “aged” sarcomeres. Further research in experimental model systems is needed to determine if targeting tubulin and protein quality control at an early disease stage prevent the progression of cardiac dysfunction.

## Data Availability Statement

The datasets presented in this study can be found in online repositories. The names of the repository/repositories and accession number(s) can be found below: ProteomeXchange consortium, identifier PXD012467, http://www.ebi.ac.uk/pride/archive/projects/PXD012467.

## Ethics Statement

The studies involving human participants were reviewed and approved by Local medical ethics review committee of the Erasmus Medical Center, Rotterdam, Netherlands. Written informed consent to participate in this study was provided by the participants' legal guardian/next of kin.

## Author Contributions

MS, DK, and JV conceived, designed, and coordinated the study and wrote the manuscript. MS, LD, JK, TP, TS, and SP performed and/or analyzed the experiments. CR and MM were involved in patient data and material acquisition. CJ provided supervision for proteomics experiments. All authors proof-read the manuscript and gave valuable input.

## Conflict of Interest

The authors declare that the research was conducted in the absence of any commercial or financial relationships that could be construed as a potential conflict of interest.
