# Supplementary material for: Sex-Related Differences in Protein Expression in Sarcomere Mutation-Positive Hypertrophic Cardiomyopathy
Source: Front Cardiovasc Med. 2021 Mar 1;8:612215. doi: 10.3389/fcvm.2021.612215 (PMC7956946; doi:10.3389/fcvm.2021.612215)
Supplement: Supplementary file 1 [file Data_Sheet_1.DOCX]

Supplementary Material

## Supplementary Figures


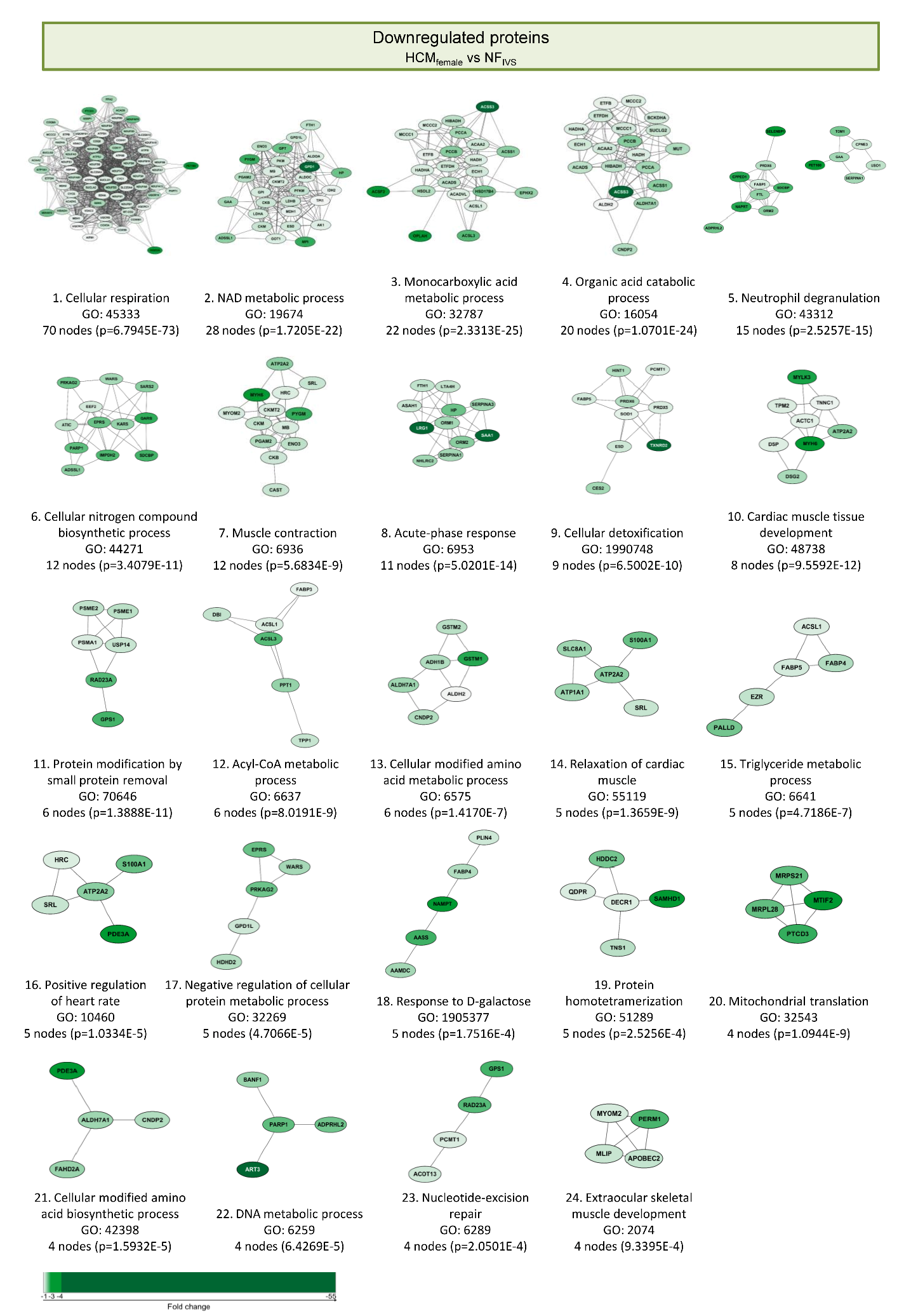


**Supplementary Figure 1.** Downregulated biological processes in HCM_female_ compared to NF_IVS_. Protein interaction cluster of significantly different downregulated proteins between HCM_female_ and NF_IVS_ were identified and are displayed with the most significant corresponding gene ontology (GO) term. The color gradient from light to dark indicates an increase in fold change.

**
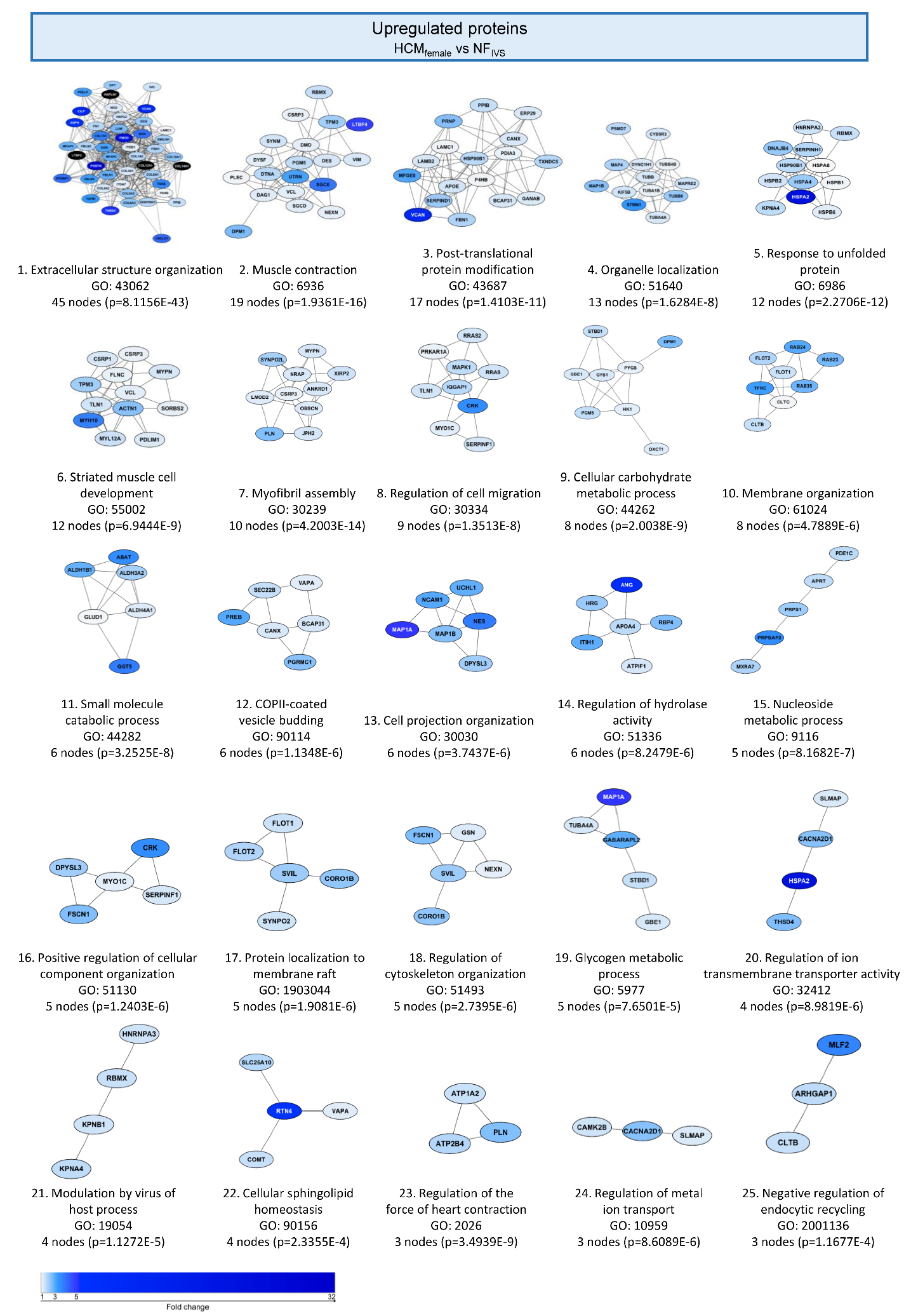
**

**Supplementary Figure 2.** Upregulated biological processes in HCM_female_ compared to NF_IVS_. Protein interaction cluster of significantly different upregulated proteins between HCM_female_ and NF_IVS_ were identified and are displayed with the most significant corresponding gene ontology (GO) term. The color gradient from light to dark indicates an increase in fold change.


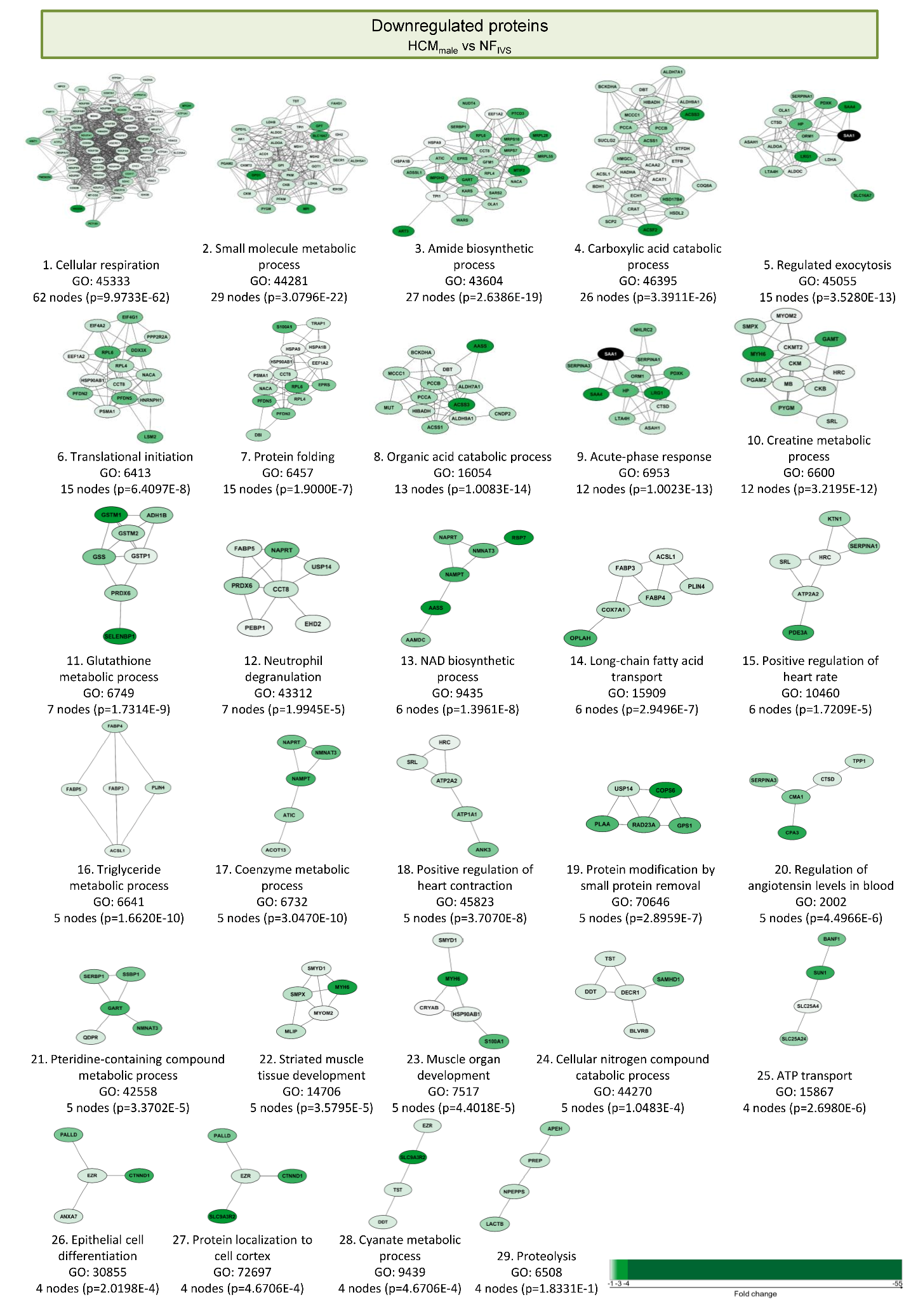


**Supplementary Figure 3.** Downregulated biological processes in HCM_male_ compared to NF_IVS_. Protein interaction cluster of significantly different downregulated proteins between HCM_male_ and NF_IVS_ were identified and are displayed with the most significant corresponding gene ontology (GO) term. The color gradient from light to dark indicates an increase in fold change.


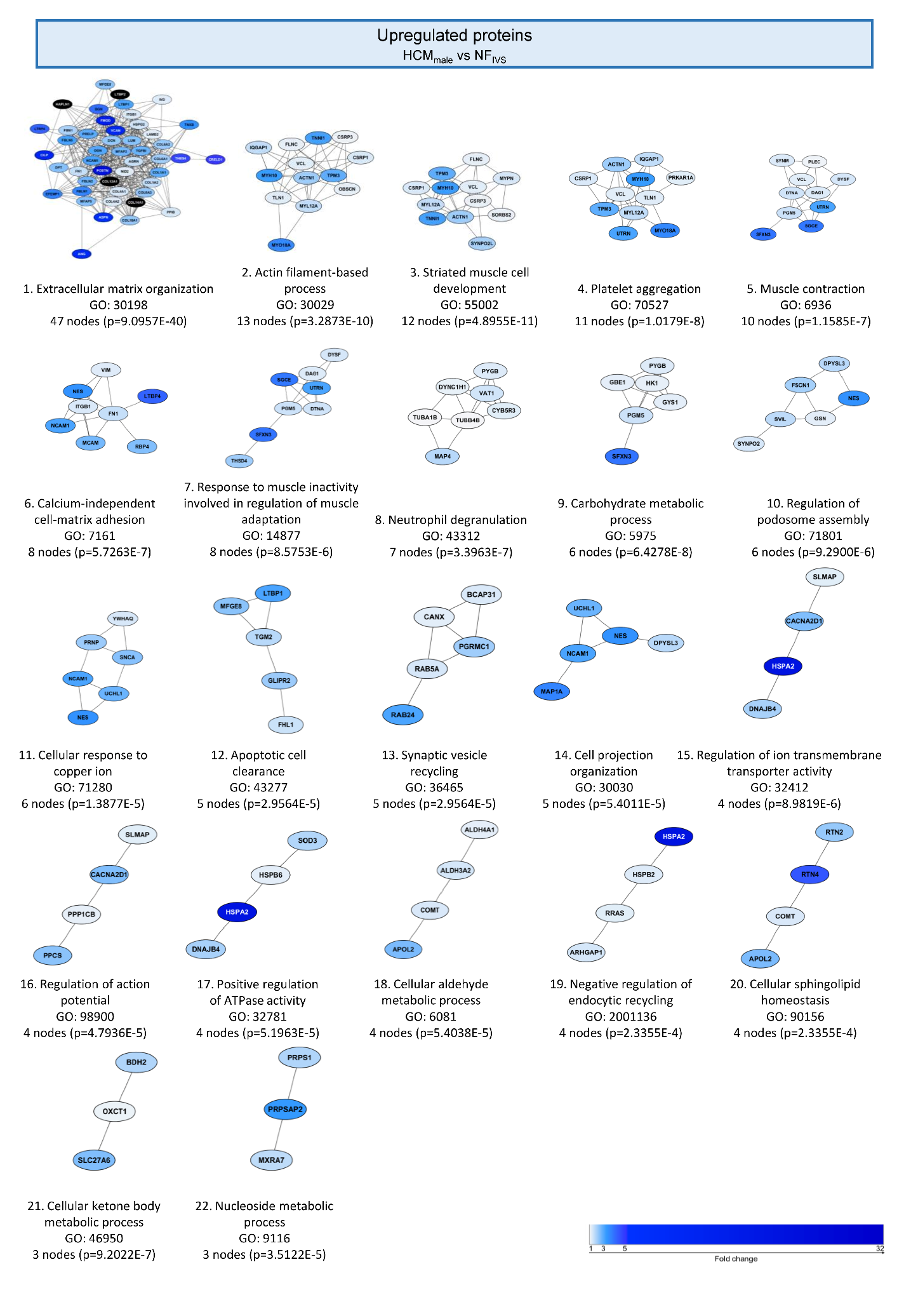


**Supplementary Figure 4.** Upregulated biological processes in HCM_male_ compared to NF_IVS_. Protein interaction cluster of significantly different upregulated proteins between HCM_male_ and NF_IVS_ were identified and are displayed with the most significant corresponding gene ontology (GO) term. The color gradient from light to dark indicates an increase in fold change.

**
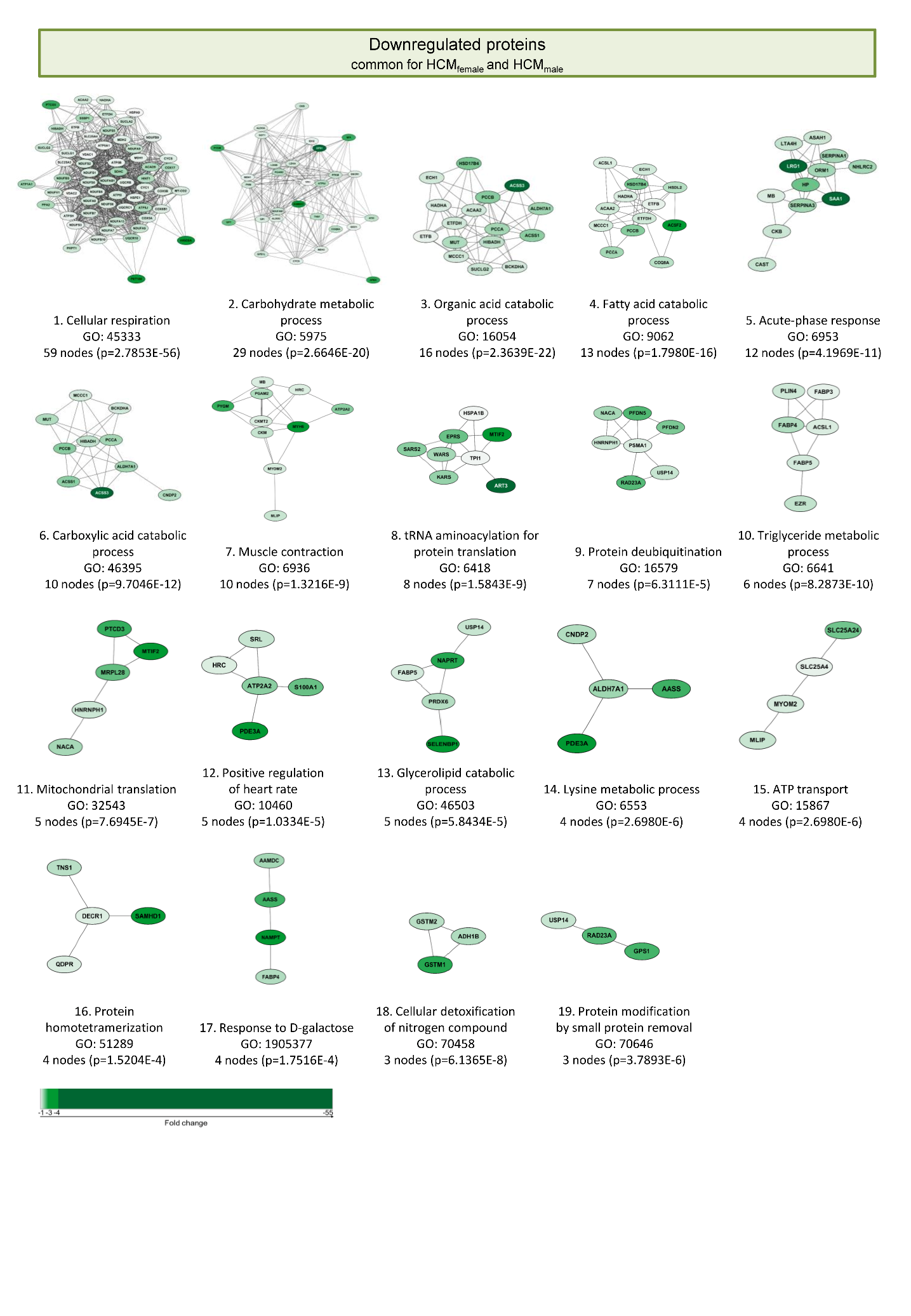
**

**Supplementary Figure 5.** Downregulated biological processes that are common for HCM_female_ and HCM_male_ when compared to NF_IVS_. Protein interaction cluster of downregulated proteins that are significantly different in both HCM_female_ and HCM_male_ when compared to NF_IVS_ (derived from Venn diagram in Figure 5) were identified and are displayed with the most significant corresponding gene ontology (GO) term. The color gradient from light to dark indicates an increase in fold change.

**
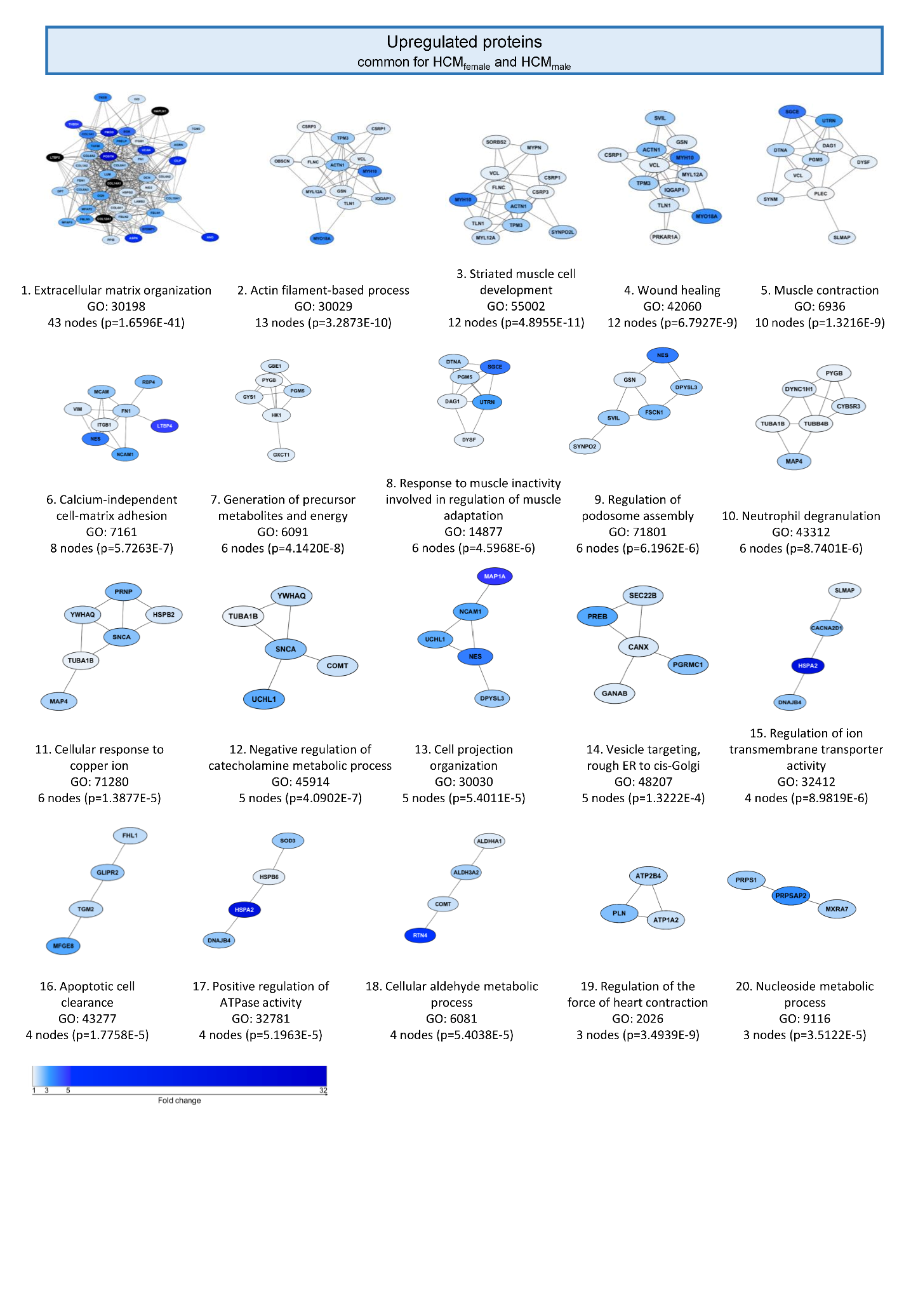
**

**Supplementary Figure 6.** Upregulated biological processes that are common for HCM_female_ and HCM_male_ when compared to NF_IVS_. Protein interaction cluster of upregulated proteins that are significantly different in both HCM_female_ and HCM_male_ when compared to NF_IVS_ (derived from Venn diagram in Figure 6) were identified and are displayed with the most significant corresponding gene ontology (GO) term. The color gradient from light to dark indicates an increase in fold change.

**Supplementary Tables**

**Table S1: Patient characteristics.** Clinical characteristics and gene mutations with mutation type (missense (mis), truncation (trunc) or deletion (del)) of all individual patients. Furthermore it presents the left ventricular (LV) parameters left atrial diameter (LAD), interventricular septum thickness (IVS), indexed interventricular septum thickness (IVS_i_), which is corrected for body surface area, end-diastolic diameter (EDD), end-systolic diameter (ESD) and fractional shortening (FS) as well as the diastolic parameters E/A ratio, E/e‘ ratio and TR velocity. The table also displays the stadium of diastolic dysfunction, left ventricular outflow tract obstruction gradient (LVOTO) and information on the patient’s medication (beta-blocker (bb), calcium channel blocker (ccb), serotonin-norepinephrine reuptake inhibitor (snri), oral anti-coagulant (oac), diuretics, angiotensin-converting enzyme inhibitor (ACEi), valproic acid (vpa), proton pump inhibitor (ppi), noradrenergic and specific serotonergic antidepressant (NaSSA), selective serotonin reuptake inhibitor (ssri), acetylsalicylic acid (asa), non-vitamin k oral anticoagulant (noac), paracetamol (pcm), angiotensin-II receptor inhibitor (ATII-i). Dark grey row below each group shows mean±SD per group.

| **Group** | **HCM ID** | **Sex** | **Age at surgery (yrs)** | **Mutation** | | | |  | **LV parameters** | | | | | | | | | **Systolic parameters** | | | | | **Diastolic parameters** | | | | **Diastolic dysfunction** | **Medication** | | | | |  |  |
| --- | --- | --- | --- | --- | --- | --- | --- | --- | --- | --- | --- | --- | --- | --- | --- | --- | --- | --- | --- | --- | --- | --- | --- | --- | --- | --- | --- | --- | --- | --- | --- | --- | --- | --- |
|  |  |  |  | **Code** | | **Type** | | LVOTO (mmHg) | LAD (mm) | IVS (mm) | IVS_i_ | EDD (mm) | | ESD (mm) | | |  | FS (%) | | | | | E/A ratio | E/e' ratio | | TR velocity (cm/s) | stage (1-4) |  | | | | |  |  |
| Female | 27 | F | 58 | *MYH7* c.4130C>T/p.T1377M | | Mis | | 100 | 48 | 20 | 13 |  | |  | | |  |  | | | | |  |  | | 2.80 |  | bb | | | | |  |  |
|  | 34 | F | 47 | *MYBPC3* c.1790G>A/p.R597Q | | Trunc | | 38 | 46 | 20 | 10 | 42 | | 18 | | |  | 57 | | | | | 2.00 | 18.30 | | 2.99 | 3 | bb, ccb, statin | | | | |  |  |
|  | 42B | F | 46 | *MYH7* c.1816G>A/p.V606M | | Mis | | 77 | 51 | 20 | 9 |  | |  | | |  |  | | | | | 3.38 | 20.30 | | 2.60 | 3 | bb, ccb | | | | |  |  |
|  | 52 | F | 24 | *MYBPC3* c.2827C>T/p.R943X | | Trunc | | 34 | 44 | 24 | 14 | 35 | | 16 | | |  | 54 | | | | | 1.12 | 18.90 | | 2.50 | 2 | bb | | | | |  |  |
|  | 88 | F | 57 | *MYL2* c.401A>C/p.E134A | | Mis | |  |  |  |  |  | |  | | |  |  | | | | |  |  | |  |  |  | | | | |  |  |
|  | 113 | F | 21 | *MYBPC3* c.2434G>A,c.2827C>T/p.R943X | | Trunc | | 27 | 43 | 45 | 28 | 40 | |  | | |  |  | | | | | 1.26 | 23.10 | | 2.29 | 2 | bb, ccb | | | | |  |  |
|  | 116 | F | 53 | *MYBPC3* c.2827C>T/p.R943X | | Trunc | |  |  |  |  |  | |  | | |  |  | | | | |  |  | |  |  |  | | | | |  |  |
|  | 123 | F | 59 | *MYBPC3* c.2373insG/p.W792fs | | Trunc | | 64 | 45 | 21 |  | 55 | |  | | |  |  | | | | | 1.20 | 32.90 | | 3.12 | 2 | bb, ccb, oac, diuretics, ACEi | | | | |  |  |
|  | 131 | F | 52 | *MYH7* c.1987C>T/p.R663C | | Mis | | 41 | 52 | 21 | 10 | 43 | |  | | |  |  | | | | |  |  | | no | _ | bb, statin, asa | | | | |  |  |
|  | 132 | F | 15 | *TNNT2* c. 814C>T/p.Q272* | | Trunc | | 81 | 37 | 19 | 13 | 39 | | 16 | | |  | 59 | | | | | 2.33 | 17.50 | | 1.99 | 3 | bb | | | | |  |  |
|  | 163 | F | 64 | *TNNI3* c.433C>T/p.R145W | | Mis | | 125 | 46 | 23 | 12 | 42 | |  | | |  |  | | | | | 0.52 | 16.30 | | no | 1 | bb, asa, thyroxine | | | | |  |  |
|  | 166 | F | 66 | *MYH7* c.2080C>T/p.R694C | | Mis | | 41 | 41 | 16 |  |  | |  | | |  |  | | | | | 0.79 | 32.40 | | no | 2 | bb, ccb, asa, pregabaline | | | | |  |  |
|  | 170 | F | 69 | *TNNI3* c.433C>T/p.R145W | | Mis | | 36 | 47 | 22 |  | 41 | |  | | |  |  | | | | | 0.78 | 36.80 | | 2.02 | 2 | bb, ccb, oac, ppi, diuretic, pcm | | | | |  |  |
|  |  | 0% male | 49±18 |  | |  | | 60±32 | 45±4 | 23±8 | 14±6 | 42±6 | | 17±1 | | |  | 57±3 | | | | | 1.49±0.92 | 24.06±7.81 | | 2.54±0.42 | 2±1 |  | | | | |  |  |
| Male | 36 | M | 22 | *MYBPC3* c.927-2A>G | | Trunc | | 71 | 60 | 30 |  | 44 | | 19 | | |  | 57 | | | | | 0.73 | 16.00 | | no | 1 | bb, ccb | | | | |  |  |
|  | 43 | M | 60 | *MYBPC3* c.2373insG/p.W792fs | | Trunc | | 77 | 52 | 23 |  | 45 | | 29 | | |  | 36 | | | | | 2.00 | 14.50 | | _ | 3 | bb, ccb | | | | |  |  |
|  | 55 | M | 46 | *TNNI3* c.433C>T/p.R145W | | Mis | | 100 | 64 | 23 | 11 | 42 | | 23 | | |  | 45 | | | | | 1.09 | 26.70 | | no | 2 | bb | | | | |  |  |
|  | 71 | M | 49 | *MYBPC3* c.2827C>T/p.R943X | | Trunc | | 9 | 47 | 16 | 7 | 44 | | 29 | | |  | 34 | | | | | 0.78 | 10.20 | | 2.30 | 1 | bb | | | | |  |  |
|  | 92 | M | 66 | *MYH7* c.2685A>C/p.Q895H | | Mis | |  |  |  |  |  | |  | | |  |  | | | | |  |  | |  |  |  | | | | |  |  |
|  | 103 | M | 26 | *MYBPC3* c.2373insG/p.W792fs | | Trunc | | 13 | 47 | 20 | 9 | 47 | | 25 | | |  | 47 | | | | | 1.33 | 13.80 | | 1.40 | 1 | bb, snri | | | | |  |  |
|  | 114 | M | 69 | *MYH7* c.976G>C/p.A326P | | Mis | | 71 | 43 | 19 |  | 33 | |  | | |  |  | | | | | 0.60 | 12.90 | | 2.14 | 1 | bb, statin, asa | | | | |  |  |
|  | 120 | M | 27 | *MYBPC3* c.2373insG/p.W792fs | | Trunc | | 61 | 39 | 24 | 13 | 37 | | 19 | | |  | 49 | | | | | 1.42 | 14.00 | | 2.33 | 1 | bb | | | | |  |  |
|  | 124 | M | 53 | *MYBPC3* c.2827C>T/p.R943X | | Trunc | | 41 | 43 | 21 | 10 | 47 | |  | | |  |  | | | | | 0.64 | 13.50 | | 2.15 | 1 | bb | | | | |  |  |
|  | 133 | M | 58 | *MYBPC3* c.442G>A/p.G148R | | Mis | | 19 | 44 | 21 | 10 | 40 | | 30 | | |  | 25 | | | | | 0.86 | 12.50 | | 2.42 | 1 | bb, oac, statin, ppi, NaSSA, ssri | | | | |  |  |
|  | 169 | M | 52 | *MYBPC3* c.2373insG/p.W792fs | | Trunc | | 100 | 45 | 21 | 10 | 43 | |  | | |  |  | | | | | 0.87 | 15.90 | | 2.27 | 1 | bb, ccb, haldol, vpa | | | | |  |  |
|  | 173 | M | 58 | *TNNT2* c.832C>T/p.R278C | | Mis | | 74 | 51 | 18 |  | 40 | |  | | |  | 62 | | | | | 2.04 | 16.60 | | 2.70 | 3 | non | | | | |  |  |
|  | 175 | M | 61 | *TNNT2* c.832C>T/p.R278C | | Mis | | 31 | 46 | 16 | 8 | 50 | |  | | |  |  | | | | | 0.75 | 12.50 | | 2.25 | 1 | bb, asa, diuretics, clopidogrel, statin | | | | |  |  |
|  |  | 100% male | 50±16 |  | |  | | 56±32 | 48±7 | 21±4 | 10±2 | 43±5 | | 25±5 | | |  | 44±12 | | | | | 1.09±0.50 | 14.93±4.11 | | 2.22±0.35 | 1±1 |  | | | | |  |  |
| NF_IVS_ | 5033 | F | 48 |  | |  | |  |  |  |  |  | |  | | |  |  | | | | |  |  | |  |  |  | | | | |  |  |
|  | 5126 | F | 55 |  |  | |  | | | | | |  | |  |  | | |  |  |  |  | | |  | | | |  |  |  |  | |  |
|  | 6008 | M | 40 |  |  | |  | | | | | |  | |  |  | | |  |  |  |  | | |  | | | |  |  |  |  | |  |
|  | 6028 | F | 62 |  |  | |  | | | | | |  | |  |  | | |  |  |  |  | | |  | | | |  |  |  |  | |  |
|  | 6056 | F | 42 |  |  | |  | | | | | |  | |  |  | | |  |  |  |  | | |  | | | |  |  |  |  | |  |
|  | 7040 | M | 37 |  |  | |  | | | | | |  | |  |  | | |  |  |  |  | | |  | | | |  |  |  |  | |  |
|  | 7054 | M | 33 |  |  | |  | | | | | |  | |  |  | | |  |  |  |  | | |  | | | |  |  |  |  | |  |
|  | 8004 | F | 50 |  |  | |  | | | | | |  | |  |  | | |  |  |  |  | | |  | | | |  |  |  |  | |  |
|  |  | 38% male | 46±10 |  |  | |  | | | | | |  | |  |  | | |  |  |  |  | | |  | | | |  |  |  |  | |  |

**Table S2:** Top 10 most significant sex hormone transcription factors involved in regulation of significantly different proteins between HCM_female_ and HCM_male_

| **Rank** | **ID** | **Name** | **p-value** | **q-value FDR B&H** | **Hit Count in Query List** | **Hit Count in Genome** | **Hit in Query List** |
| --- | --- | --- | --- | --- | --- | --- | --- |
| 1 | V$SRF_Q6 | Serum response factor | 1,10E-04 | 4,08E-02 | 5 | 211 | ACAA2, DMD, PDLIM3, AGL, LRRFIP1 |
| 2 | V$E2A_Q2 | Transcription factor E2-alpha | 9,74E-04 | 1,81E-01 | 4 | 193 | CRYAB, DMD, ATP2A2, ENO3 |
| 3 | V$HSF_Q6 | Heat shock factor | 5,20E-03 | 2,61E-01 | 3 | 152 | HSPD1, CRYAB, MYH14 |
| 4 | V$HNF3_Q6 | Hepatocyte nuclear factor 3 | 6,00E-03 | 2,61E-01 | 3 | 160 | MACROH2A1, DMD, ATP2A2 |
| 5 | CTGRYYYNATT_UNKNOWN |  | 6,79E-03 | 2,61E-01 | 2 | 53 | CRYAB, DMD |
| 6 | V$HFH4_01 | Forkhead Box J1 | 6,98E-03 | 2,61E-01 | 3 | 169 | MACROH2A1, DMD, ATP2A2 |
| 7 | V$FOX_Q2 | Forkhead Box | 8,05E-03 | 2,61E-01 | 3 | 178 | MACROH2A1, DMD, ATP2A2 |
| 8 | CCGNMNNTNACG_UNKNOWN |  | 8,92E-03 | 2,61E-01 | 2 | 61 | HSPD1, CRK |
| 9 | V$NKX3A_01 | Homeobox Protein NK-3 Homolog A | 8,95E-03 | 2,61E-01 | 3 | 185 | TPM1, DMD, ATP2A2 |
| 10 | V$COUP_DR1_Q6 | chicken ovalbumin upstream promoter | 1,02E-02 | 2,61E-01 | 3 | 194 | HSPD1, DMD, ENO3 |

**Table S3:** Sex hormone transcription factors involved in regulation of significantly different proteins between HCM_female_ and HCM_male_

| **Rank** | **ID** | **Name** | **p-value** | **q-value FDR B&H** | **Hit Count in Query List** | **Hit Count in Genome** | **Hit in Query List** |
| --- | --- | --- | --- | --- | --- | --- | --- |
| 32 | TGACCTY_V$ERR1_Q2 | Estrogen-related receptor alpha | 4,14E-02 | 3,47E-01 | 5 | 834 | HSPD1,PYGM,DMD,ENO3,TFRC |
| 85 | V$ERR1_Q2 | Estrogen-related receptor alpha | 8,66E-02 | 3,47E-01 | 2 | 210 | HSPD1,ENO3 |
| 112 | V$AR_03 | Androgen receptor | 1,11E-01 | 3,70E-01 | 1 | 50 | PYGM |
| 145 | V$AR_Q2 | Androgen receptor | 2,13E-01 | 4,49E-01 | 1 | 101 | DMD |
| 151 | V$PR_02 | Progesterone receptor | 2,31E-01 | 4,49E-01 | 1 | 111 | PYGM |
| 156 | V$PR_01 | Progesterone receptor | 2,42E-01 | 4,49E-01 | 1 | 117 | PYGM |
| 236 | V$AR_Q6 | Androgen receptor | 3,70E-01 | 4,49E-01 | 1 | 194 | DMD |
